# HOME CARE DYADIC COMMUNICATION PATTERNS OF FAMILY CAREGIVERS AND PERSONS LIVING WITH DEMENTIA: SEQUENTIAL ANALYSIS

**DOI:** 10.1093/geroni/igad104.0816

**Published:** 2023-12-21

**Authors:** Sohyun Kim, Wen Liu, Kristine Williams

**Affiliations:** University of Texas at Arlington, Arlington, Texas, United States; The University Of Iowa, Iowa City, Iowa, United States; University of Kansas Medical Center, Kansas City, Kansas, United States

## Abstract

Examining temporal relationships of communication patterns between family caregivers and persons living with dementia (care recipients) is useful to determine which caregiver communication patterns are more effective in engaging care recipients and prioritize the use of such communication patterns. This study examined temporal relationships between antecedent family caregiver communication and subsequent care recipient communication during 75 in-home care video observations. A 5-second timed-window analysis was conducted to examine the likelihood of the relationships between dyadic communication patterns. Joint frequency and conditional probability statistics demonstrated that care recipients used the same communication patterns at differing frequencies during and within a 5-second window following each communication pattern by family caregivers. Dyadic communication patterns yielded likelihood probabilities with an effect size greater than chance (Yule’s Q = 0.31-1.00). Care recipient’s engaging body language most likely occurred after the caregiver’s facilitative body language preceded (Yule’s Q = 0.31-.1.00). Care recipient’s relevant communication and verbal response most likely occurred after the caregiver’s simplified message preceded (Yule’s Q = 0.55-1.00). Specific caregiver facilitative communication exhibited subsequent care recipient engaging communication. Future research can expand on how characteristics, types, and frequencies of caregiver communication would impact care recipient engaging communication.

